# Gene expression analysis of resistant and susceptible rice cultivars to sheath blight after inoculation with *Rhizoctonia solani*

**DOI:** 10.1186/s12864-022-08524-6

**Published:** 2022-04-07

**Authors:** Xiaohe Yang, Xin Gu, Junjie Ding, Liangliang Yao, Xuedong Gao, Maoming Zhang, Qingying Meng, Songhong Wei, Junfan Fu

**Affiliations:** 1 College of Plant Protection, Shenyang Agricultural University, Shenyang, 110161 Liaoning China; 2 Jiamusi Branch of Heilongjiang Academy of Agricultural Sciences, Jiamusi, 154007 Heilongjiang China

**Keywords:** Japonica rice, Rice sheath blight, Transcriptome, Defence response, Host-pathogen interaction

## Abstract

**Background:**

Rice sheath blight, caused by *Rhizoctonia solani* Kühn (teleomorph: *Thanatephorus cucumeris*), is one of the most severe diseases in rice (*Oryza sativa* L.) worldwide. Studies on resistance genes and resistance mechanisms of rice sheath blight have mainly focused on indica rice. Rice sheath blight is a growing threat to rice production with the increasing planting area of japonica rice in Northeast China, and it is therefore essential to explore the mechanism of sheath blight resistance in this rice subspecies.

**Results:**

In this study, RNA-seq technology was used to analyse the gene expression changes of leaf sheath at 12, 24, 36, 48, and 72 h after inoculation of the resistant cultivar ‘Shennong 9819’ and susceptible cultivar ‘Koshihikari’ with *R. solani*. In the early stage of *R. solani* infection of rice leaf sheaths, the number of differentially expressed genes (DEGs) in the inoculated leaf sheaths of resistant and susceptible cultivars showed different regularity. After inoculation, the number of DEGs in the resistant cultivar fluctuated, while the number of DEGs in the susceptible cultivar increased first and then decreased. In addition, the number of DEGs in the susceptible cultivar was always higher than that in the resistant cultivar. After inoculation with *R. solani*, the overall transcriptome changes corresponding to multiple biological processes, molecular functions, and cell components were observed in both resistant and susceptible cultivars. These included metabolic process, stimulus response, biological regulation, catalytic activity, binding and membrane, and they were differentially regulated. The phenylalanine metabolic pathway; tropane, piperidine, and pyridine alkaloid biosynthesis pathways; and plant hormone signal transduction were significantly enriched in the early stage of inoculation of the resistant cultivar Shennong 9819, but not in the susceptible cultivar Koshihikari. This indicates that the response of the resistant cultivar Shennong 9819 to pathogen stress was faster than that of the susceptible cultivar. The expression of plant defense response marker *PR1b* gene, transcription factor *OsWRKY30* and *OsPAL1* and *OsPAL6* genes that induce plant resistance were upregulated in the resistant cultivar. These data suggest that in the early stage of rice infection by *R. solani*, there is a pathogen-induced defence system in resistant rice cultivars, involving the expression of *PR* genes, key transcription factors, *PAL* genes, and the enrichment of defence-related pathways.

**Conclusion:**

The transcriptome data revealed the molecular and biochemical differences between resistant and susceptible cultivars of rice after inoculation with *R. solani*, indicating that resistant cultivars have an immune response mechanism in the early stage of pathogen infection. Disease resistance is related to the overexpression of *PR* genes, key transcriptome factors, and *PAL* genes, which are potential targets for crop improvement.

**Supplementary Information:**

The online version contains supplementary material available at 10.1186/s12864-022-08524-6.

## Background

Rice (*Oryza sativa* L.) is one of the three most important crops worldwide, and rice sheath blight is one of the most destructive diseases [1–3]. The annual loss of rice products caused by rice sheath blight is as high as 50% worldwide [4–7]. *Rhizoctonia solani* is a soil-borne fungal plant pathogen [8]. The host range of the pathogen is wide, and the sclerotium of the pathogen is strongly resistant to the external environment [9, 10]. Due to the lack of resistant donors in cultivated cultivars [11, 12], studies on the resistance mechanism of rice sheath blight are lacking [13, 14]. For a long time, studies on the resistance genes and mechanisms underlying rice sheath blight have focused mainly on indica rice [15–17]. However, the total area of japonica rice in China accounts for one-third of the total area of rice plantations in China. Furthermore, the japonica rice planting area in Northeast China accounts for 53.7% of the total area of japonica rice [18]. In recent years, sheath blight has severely affected the production of japonica rice in Northeast China [19]. Koshihikari, a high-quality japonica rice cultivar, was developed in Japan in 1956. It is well known for its high eating quality [20]. At the end of July 2007, Japanese Koshihikari brand rice entered the Chinese market, with a price of up to 99 yuan Renminbi per kg, which attracted the attention of Chinese rice breeders to high-quality rice breeding [21]. Although the Koshihikari cultivar has poor disease resistance and is susceptible to lodging, it is still an excellent parent for high-quality rice breeding. In this study, the susceptible cultivar Koshihikari and the resistant cultivar Shennong 9819 were used to analyse the early transcripts of *R. solani* infection and explore its disease resistance mechanism.

With the rapid development of molecular biology techniques and the wide application of various omics technology in the interaction between plants and pathogens, the identification of rice sheath blight resistance genes and the interaction mechanism between sheath blight pathogen and rice are becoming increasingly deep. Chitinases are the members of PR proteins responsible for the hydrolysis of chitin, a structural polysaccharide of the cell wall of many pathogens. Overexpression of the chitinase gene *CHI11* enhanced resistance to rice sheath blight [22, 23]. *OsOSM1*, a gene mainly expressed in the leaf sheath at the booting stage in rice, encodes an osmotin protein belonging to the pathogenesis-related protein 5 family. Overexpression of this gene can enhance the resistance of rice to sheath blight [24]. Plant polygalacturonase-inhibiting protein (PGIP) is a structural protein that specifically recognise and bind to fungal polygalacturonase (PG). PGIP plays an important role in antifungal activity in plants. Overexpression of PGIP-related genes such as *ZmPGIP3*, *OsPGIP1*, and *OsPGIP2* increases resistance to rice sheath blight in rice [25–27]. Lignin deposition can enhance plant cell walls against pathogens and provide structural barriers for pathogen infection [28]. Overexpression of the lignin-related gene *OsPAL4* increases resistance to rice sheath blight [29]. OsWRKY4 is an important positive regulatory factor in the interaction between rice and pathogens. It participates in the defence response of rice sheath blight through the jasmonic acid (JA)/ethylene (ET)-dependent signalling pathway [30].

Due to its high throughput and sensitivity, RNA-sequencing (RNA-Seq) technology is increasingly being used in the research and analysis of gene function [31, 32]. Transcriptome sequencing technology has been successfully used to study plant–pathogen interactions [33, 34]. Bagnaresi et al. [35] used comparative transcriptome technology to analyse the early molecular interaction of resistant and susceptible rice cultivars infected with *Magnaporthe grisea.* They found that chitinase and WRKY transcription factors were involved in the resistance of rice blast. Strauss et al. [36] identified one major Bs4C candidate transcript from pepper by RNA-seq to regulate the transcription activator-like effector AvrBs4 of *Xanthomonas*. Kawahara et al. [37] analysed the mixed transcripts of rice and blast fungus in infected leaves at 24 h after inoculation using the RNA-Seq technique. It was found that in the interaction between host plants and pathogens, the transcripts of glycosyl hydrolase, cutinases, and LysM domain-containing proteins of *M. grisea* were up-regulated, including the pathogenesis-related and phytoalexin biosynthetic genes in rice. Xiao et al. used next-generation sequencing technology to study the gene expression profiles of *Fusarium* head blight-related genes in common wheat. It was found that pathogen-related proteins such as *PR5*, *PR14*, *ABC transporter*, and JA signalling pathway were the key to *Fusarium* head blight resistance [38].

Transcriptional changes in indica rice cultivar inoculated with *R. solani* were analysed using RNA-Seq technology [39]. The rice cultivar resistant to sheath blight used in our study was japonica rice cultivar from Northeast China. This is the first study to compare the gene expression patterns of resistant and susceptible japonica rice inoculated with *R. solani*. In this study, the transcripts of resistant and susceptible cultivars were compared at 12, 24, 36, 48, and 72 h after inoculation with *R. solani*. The results showed significant differences in the expression of differentially expressed genes and genes related to metabolic pathways. The expression characteristics of metabolic pathways related to disease resistance were defined. The key genes related to rice sheath blight resistance were identified, which provided gene resources for molecular-assisted breeding of japonica rice in Northeast China.

## Results

### Symptoms of leaf sheath after inoculation

The symptoms of leaf sheath after inoculation are shown in Fig. S1. After inoculation for 24 h, evident brown spots appeared on the leaf sheath of Koshihikari; 48 h after inoculation, they were grey. These spots appeared on the leaf sheath of Shennong 9819 at 36 h after inoculation. The expanded area of the spot of Koshihikari was significantly larger than that of Shennong 9819 at 72 h after inoculation.

The inoculated leaf sheath was decolorized by chloral hydrate, stained with aniline blue, and observed under a light microscope. At 12 h after inoculation, hyphae were observed in the leaf sheaths of both cultivars, and more hyphae were found in the leaf sheath of Koshihikari than in Shennong 9819. At 24 h after inoculation, infection cushions appeared in the leaf sheaths of both cultivars. The number and density of the infection cushions of Koshihikari were greater than those of Shennong 9819 (Fig. S2).

### RNA-seq results of Transcriptome samples

To study the changes in gene expression of the leaf sheath of Shennong 9819 and Koshihikari at the initial infection stage of *R. solani*, we used high-throughput sequencing technology to measure the transcription in rice leaf sheaths after inoculation. The transcriptome analysis of each cultivar included five time points with three biological repeats at each time point. A total of 342.24 Gb clean data was obtained from 36 samples; the clean data of each sample reached 8.08 Gb, and the percentage of Q30 base was 92.49% or more (Table S1). A total of 2,294,868,004 single-end clean reads (total records) were obtained after pre-processing the reads (Table S2). The clean reads of each sample were sequenced with the designated reference genome, and the efficiency of alignment ranged from 80.43 to 92.33%. The correlation analysis among the samples showed that the three repeats of the two cultivars had a high correlation (Fig. S3).

### Differential gene analysis of leaf sheath after inoculation

To determine which gene expression had changed and the stage of these changes, we counted the number of different genes between the two rice cultivars at each time point after inoculation with *R. solani* (Table 1) (SS for Shennong 9819; YY for Koshihikari). A total of 2275 differentially expressed genes (DEGs) were identified in our study (Table S3). After inoculation with *R. solani*, the number of upregulated genes in the leaf sheath of Koshihikari was higher than that of Shennong 9819 at all inoculation time points.

**Table 1:** Statistics of differentially expressed genes

DEG Set	DEG Number	up-regulated	down-regulated
SS12	2403	1242	1161
SS24	190	129	61
SS36	2127	815	1312
SS48	790	658	132
SS72	1120	818	302
YY12	2817	1419	1398
YY24	3392	1983	1409
YY36	4873	2438	2435
YY48	1303	661	642
YY72	1766	881	885

At 12 h, 2403 DEGs (1242 upregulated and 1161 downregulated) were identified in the leaf sheath of Shennong 9819, whereas 2817 DEGs (1419 upregulated and 1398 downregulated) were identified in Koshihikari, indicating that the Koshihikari was more susceptible to *R. solani* than Shennong 9819; the infection pressure on Koshihikari plants was thus higher than that on Shennong 9819. From 12 to 72 h, the number of DEGs in the Koshihikari leaf sheath was higher than Shennong 9819. The number of DEGs in the Koshihikari leaf sheath was highest at 36 h (4,873 DEGs; 2438 upregulated, 2435 downregulated) after inoculation. The data show that the number of DEGs in the susceptible cultivar was higher than that in the resistant cultivar.

The DEGs at 12, 24, 36, 48, and 72 h after inoculation of *R. solani* were analysed for the two cultivars. The DEGs related to the infection response of *R. solani* in rice were further analysed.

**Fig. 1:**
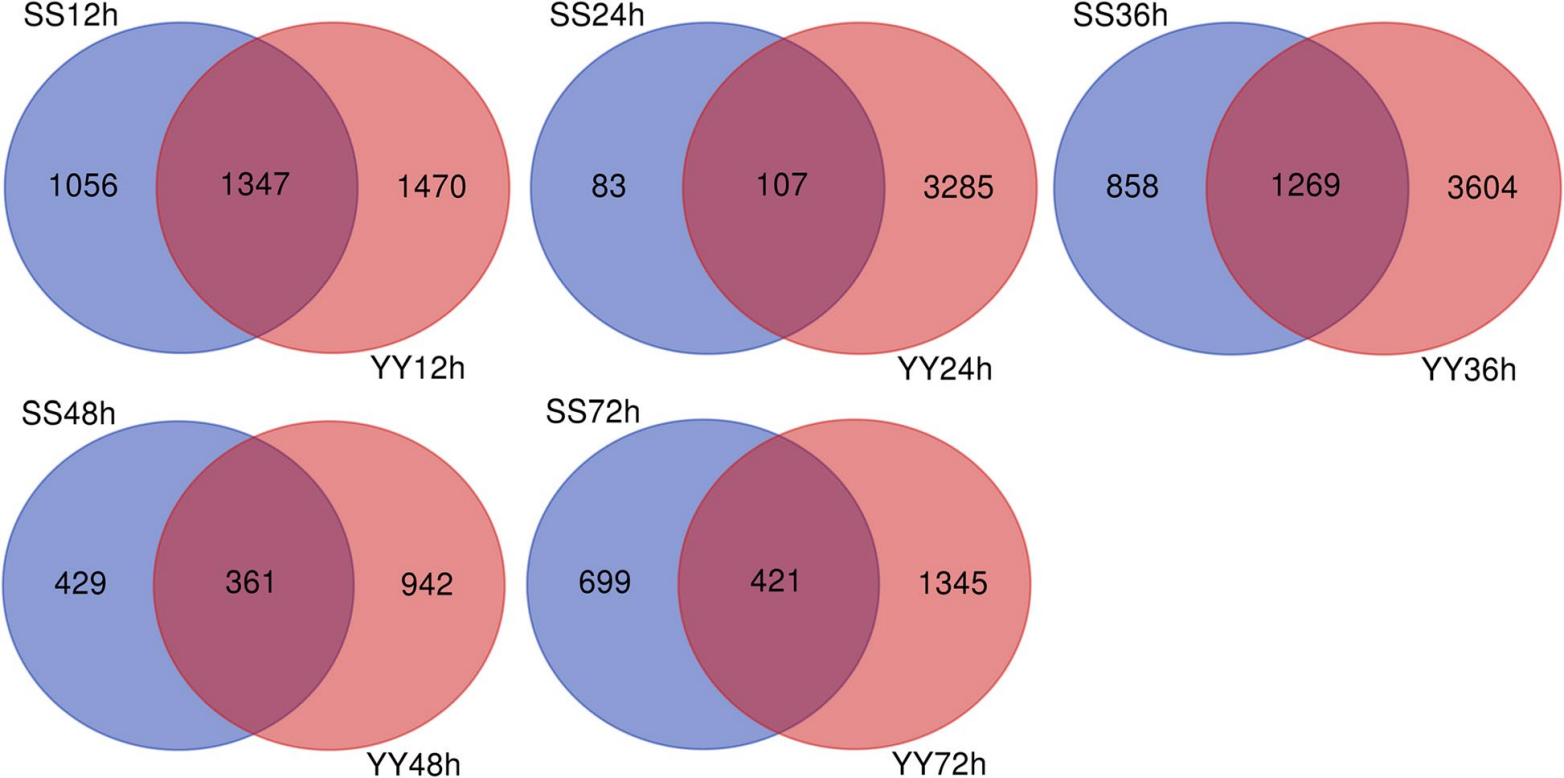
Venn diagram of DEGs discovered in both rice cultivars after inoculation with *Rhizoctonia solani* at the same time points. SS12, SS24, SS36, SS48, and SS72 represent DEG sets discovered from the leaf sheath of Shennong 9819 at 12, 24, 36, 48, and 72 h after inoculation; YY12, YY24, YY36, YY48, and YY72 represent DEG sets discovered from the leaf sheath of Koshihikari at 12, 24, 36, 48, and 72 h after inoculation

**Fig. 2:**
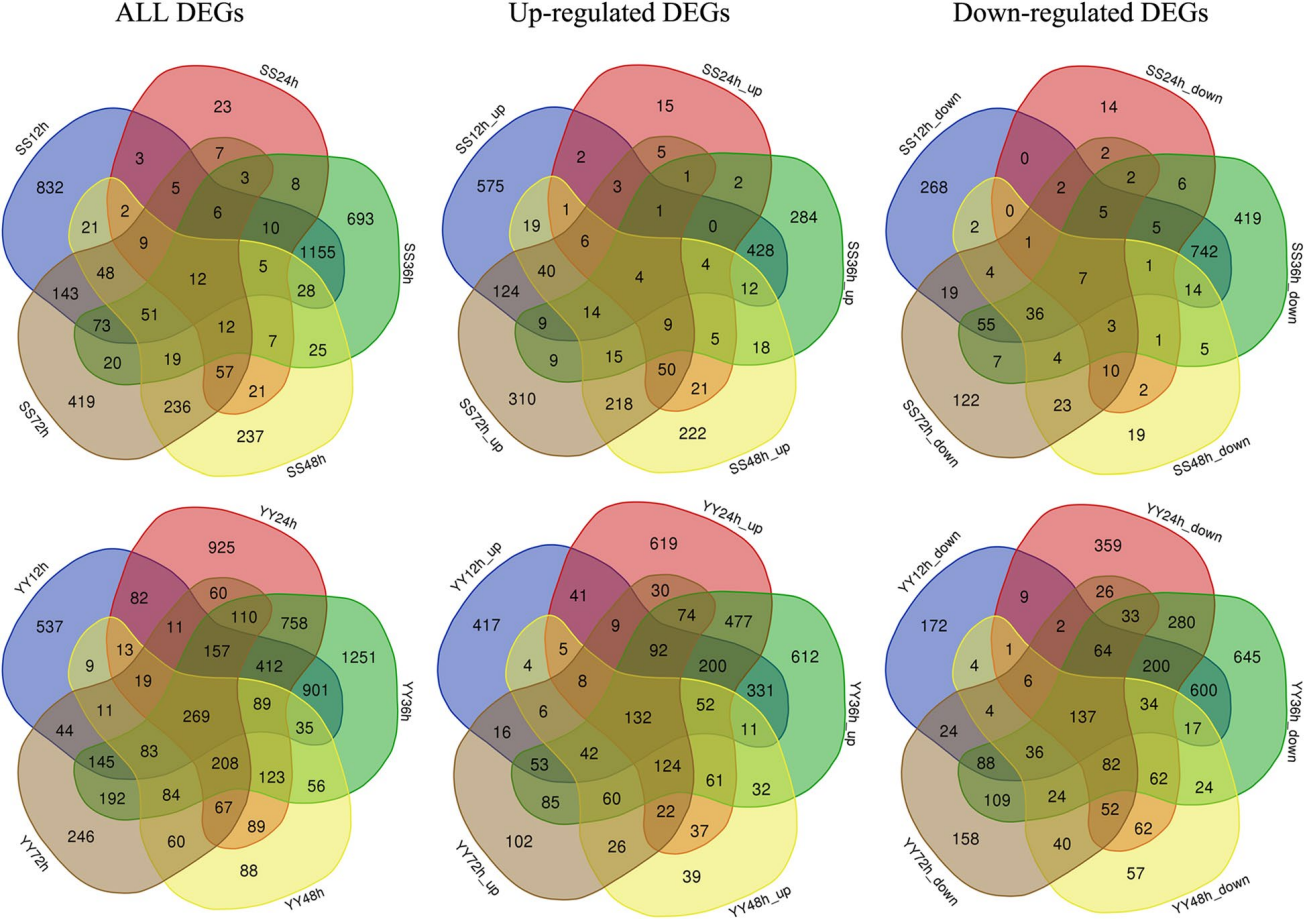
Venn diagram of DEGs in both rice cultivars after inoculation with *Rhizoctonia solani* at the different time points. SS12, SS24, SS36, SS48, and SS72 represent DEG sets discovered from the leaf sheath of Shennong 9819 at 12, 24, 36, 48, and 72 h after inoculation; YY12, YY24, YY36, YY48, and YY72 represent DEG sets discovered from the leaf sheath of Koshihikari at 12, 24, 36, 48, and 72 h after inoculation

In this study, the DEGs of the two cultivars at the same time point (SS12-YY12, SS24-YY24, SS36-YY36, SS48-YY48, and SS72-YY72) were compared (Fig. 1), and the number of DEGs for each cultivar at different time points (SS12-SS24-SS36-SS48-SS72 or YY12-YY24-YY36-YY48-YY72h) were also compared (Fig. 2). As shown in Fig. 1, 1,347 and 1269 DEGs were identified in the two cultivars at 12 h and 36 h after inoculation, respectively, and the number of these DEGs was higher than that at other time points.

In the leaf sheaths of Shennong 9819, 12 common DEGs were continuously expressed at 12, 24, 36, 48, and 72 h after inoculation, including four upregulated genes and seven downregulated genes. At 12 h, 832 specific DEGs were identified (575 upregulated and 268 downregulated). There were 23 specific DEGs (15 upregulated and 14 downregulated) discovered at 24 h, and 693 specific DEGs (284 upregulated and 419 downregulated) were identified at 36 h. In the leaf sheath of Koshihikari, 269 common DEGs were expressed at 12, 24, 36, 48, and 72 h after inoculation (132 upregulated and 137 downregulated). At 12 h, 537 specific DEGs (417 upregulated and 172 downregulated) were identified, and 925 specific DEGs (619 upregulated and 359 downregulated) were identified at 36 h. At 36 h, 1251 specific DEGs were identified (612 upregulated and 645 downregulated). In conclusion, the number of DEGs which were continuously expressed in the sheath of Koshihikari was higher than that in Shennong 9819 after inoculation.

### Gene Ontology (GO) analysis of differentially expressed genes

**Fig. 3:**
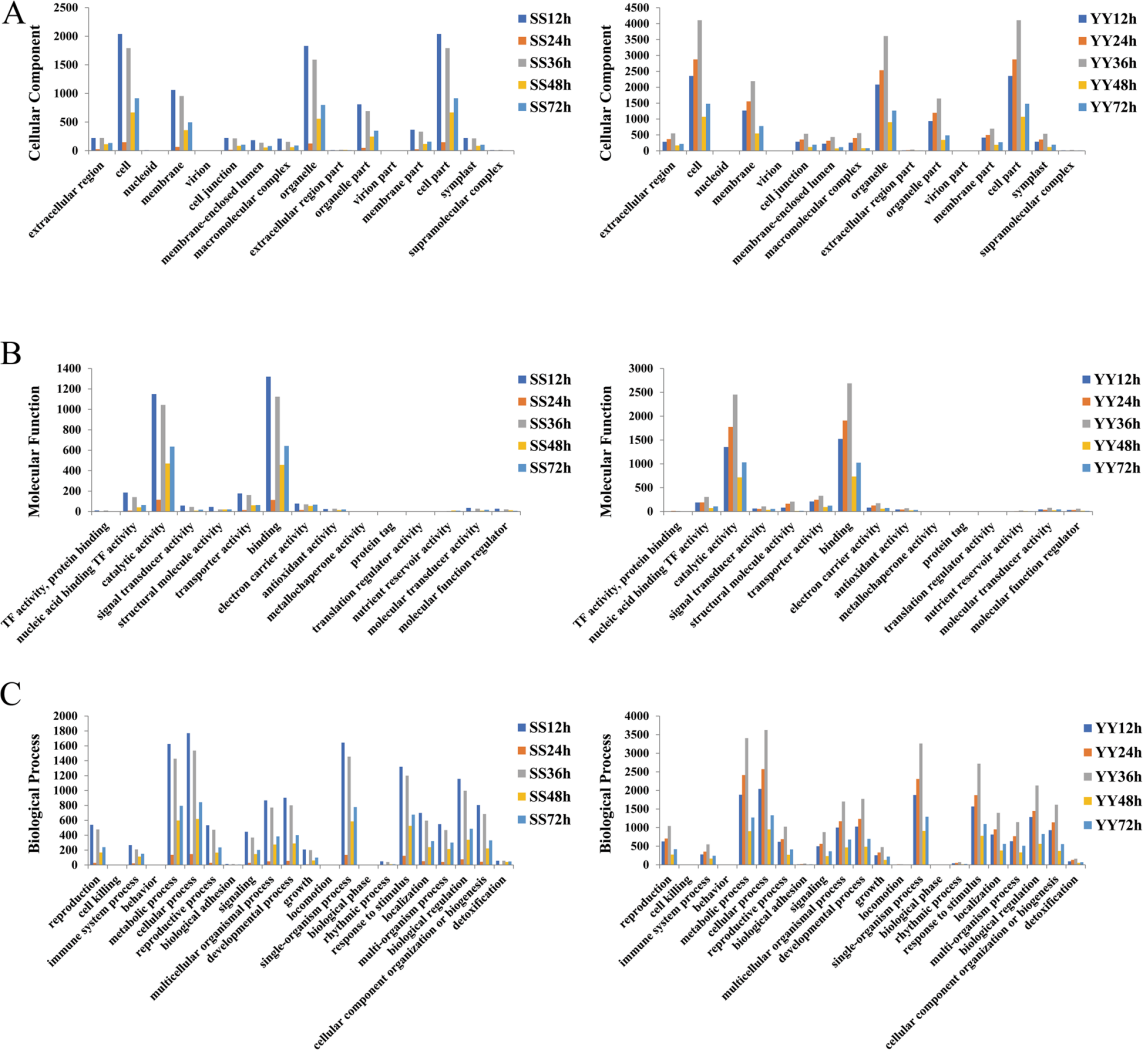
Functional classification of DEGs in the Shennong 9819 and Koshihikari. SS12, SS24, SS36, SS48, and SS72 represent DEG sets discovered from the leaf sheath of Shennong 9819 at 12, 24, 36, 48, and 72 h after inoculation; YY12, YY24, YY36, YY48, and YY72 represent DEG sets discovered from the leaf sheath of Koshihikari at 12, 24, 36, 48, and 72 h after inoculation. **A** Cellualr component **B** Molecular function **C** Biological process

GO annotation was used to classify the enriched DEGs between the control and inoculation treatments. The results showed that these enriched DEGs were involved in many biological activities (Fig. 3).

After annotating the GO database, all the DEGs were classified into three main categories: biological process, molecular function, and cellular component. In the biological process, “metabolic process”, “cellular process”, “single-organism process”, “response to stimulus”, and “biological regulation” were the five processes with the highest degree of DEGs enrichment. In terms of molecular function, most DEGs were concentrated in two processes: “catalytic activity” and “binding”. For the cellular component, the five most common processes were “cell”, “membrane”, “organelle”, “organelle part” and “cell part”.

The results showed that the GO terms in different cultivars showed completely different patterns of expression. Taking “response to stimulation” as an example, in the leaf sheath of Shennong 9819, the number of DEGs related to this process reached the maximum at 12 h. The number of DEGs was the least at 24 h after inoculation, indicating that plants responded most strongly to external stimuli at 12 h and were closest to the uninoculated state at 24 h. The results showed that the plants could repair themselves in response to external stimuli; at 36 h, the number of DEGs increased, and again at 48 h, the number of DEGs decreased. Shennong 9819 exhibited a fluctuating response to external stress. However, the number of DEGs related to the process of “response to stimulation” increased rapidly in the leaf sheath of Koshihikari after inoculation, reached a maximum at 36 h and then decreased sharply. The genes related to this process in the leaf sheath of Koshihikari were always active after inoculation. The results showed that Shennong 9819 and Koshihikari had different resistance patterns to *R. solani* infection, and the genes in the leaf sheath of Koshihikari were always in a higher activity state.

### Analysis of metabolic pathways of two rice cultivars after inoculation

To further study the specificity of pathways affected by *R. solani* infection in Shennong 9819 and Koshihikari, Kyoto Encyclopaedia of Genes and Genomes (KEGG) enrichment analysis was performed on the upregulated genes (|log2fc > 1|, FDR < 0.05) at different times after inoculation (Table S4 and S5). The results showed that alanine, aspartate and glutamate metabolism was significantly enriched in both cultivars at 12 h after inoculation, although Shennong 9819 was more significant. The phenylalanine metabolism, plant hormone signal transduction, tropane, piperidine and pyridine alkaloid biosynthesis pathways were significantly enriched in Shennong 9819 at 12 and 36 h after inoculation; however, no significant difference was found in Koshihikari. The tyrosine metabolism and isoquinoline alkaloid biosynthesis pathways were significantly enriched in Shennong 9819 at 24 and 36 h after inoculation, but not in Koshihikari. At 36 h after inoculation of Shennong 9819 and Koshihikari with *R. solani*, glycine, serine and threonine metabolism and beta-alanine metabolism pathways were significantly enriched, whereas Koshihikari was not.

Similarly, some pathways were specifically enriched in Koshihikari. For example, ascorbate and aldarate metabolism pathway was significantly enriched at 12 and 24 h after inoculation in Koshihikari. The linoleic acid metabolism pathway was significantly enriched 24 h after inoculation in Koshihikari. Valine, leucine and isoleucine degradation pathway was significantly enriched at 24 and 36 h in Koshihikari after inoculation. The arginine biosynthesis and 2-Oxocarboxylic acid metabolism pathways were significantly enriched at 36 h in Koshihikari after inoculation. The propanoate metabolism pathway was significantly enriched at 24, 36, 48, and 72 h in Koshihikari after inoculation. The above pathways were not significantly enriched at any time in Shennong 9819 after inoculation. At different times after inoculation, the upregulated genes of the two cultivars had specific enrichment pathways, suggesting that the resistance mechanisms of the two cultivars might be different.

**Fig. 4:**
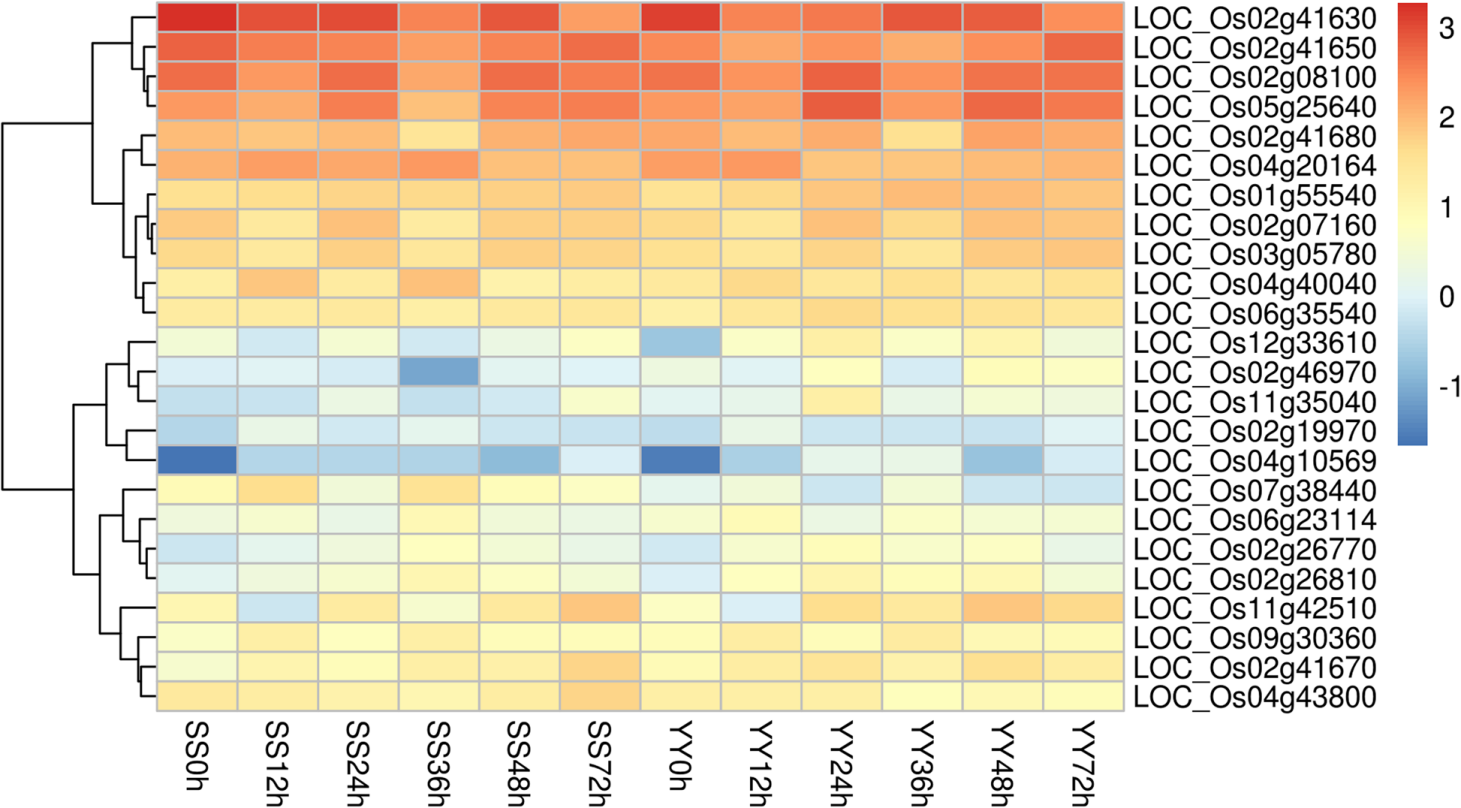
Expression profiles of genes related to phenylalanine metabolism pathways

**Fig. 5:**
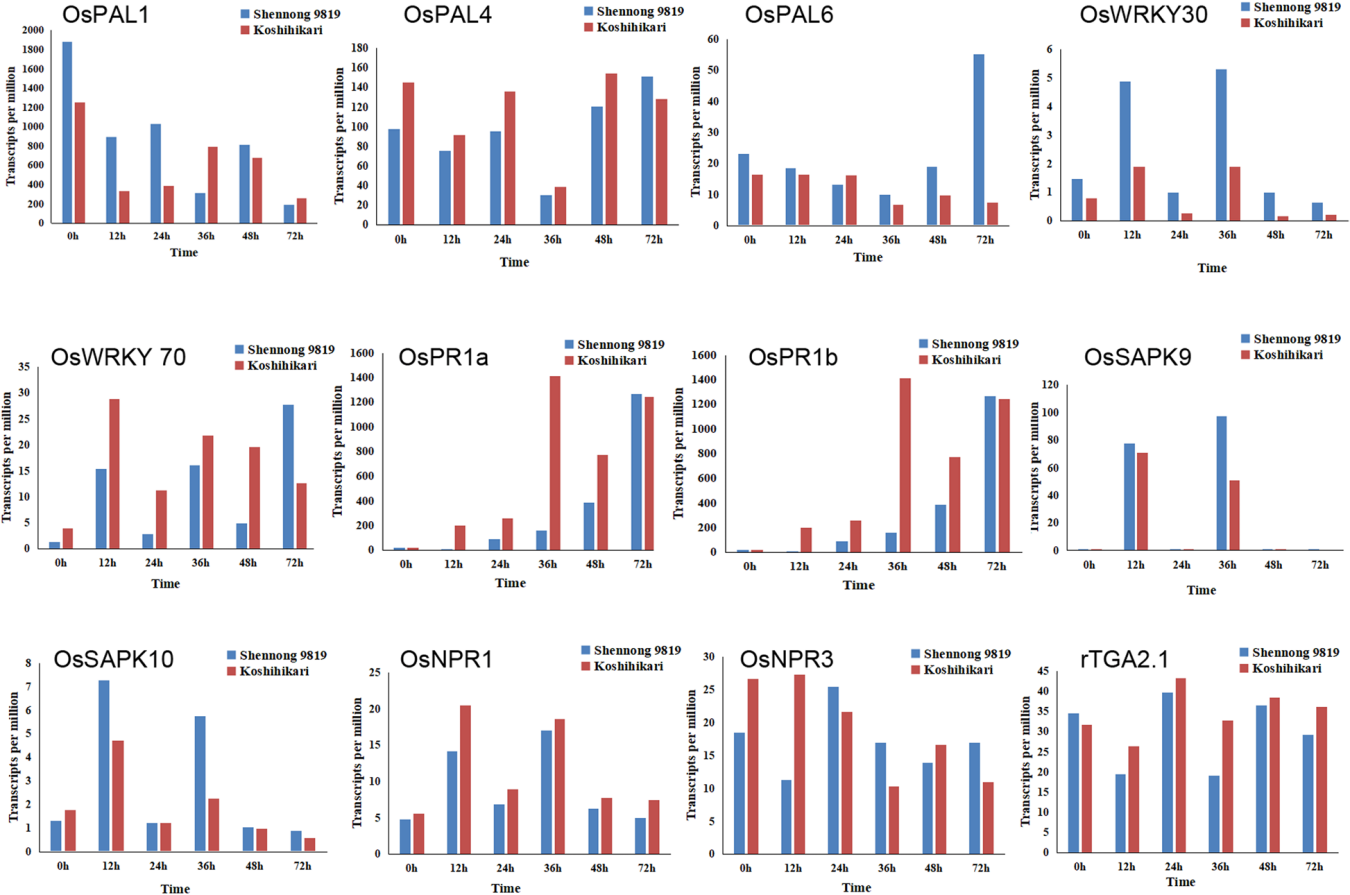
Expression of genes related to phenylalanine metabolism, plant–pathogen interaction, and plant signal transduction after inoculation based on transcripts per million (TPM)

To identify potential regulatory genes closely related to the phenylalanine metabolism pathway, DEGs involved in phenylalanine metabolism were identified by comparing the two cultivars. At 12 and 36 h, the expression levels of genes related to phenylalanine metabolism in the Shennong 9819 leaf sheath were significantly higher than those in Koshihikari. The difference between the two cultivars is shown in the heatmap (Fig. 4). Through gene analysis, a series of PAL genes was activated after inoculation with *R. solani*: *OsPAL1* (LOC_Os02g41630), *OsPAL2* (LOC_Os02g41650), *OsPAL3* (LOC_Os02g41670), *OsPAL4* (LOC_Os02g41680), *OsPAL6* (LOC_Os04g43800), and *OsPAL9* (LOC_Os12g33610). In this study, the expression of *OsPAL1* and *OsPAL6* in the leaf sheath of Shennong 9819 was higher than that in Koshihikari (Fig. 5). However, the expression of *OsPAL4* and *OsPAL9* in Koshihikari was higher than that in Shennong 9819 (Fig. 5).

We compared and analysed the genes involved in the plant–pathogen interaction pathway during early infection of Shennong 9819 and Koshihikari by *R. solani*. Pathogenesis-related proteins (PR) are defined as plant proteins that are induced in pathological or related situations. The expression of *OsPR1a* (LOC_Os07g03710) was upregulated in both resistant cultivar Shennong 9819 and susceptible cultivar Koshihikari. The expression of *OsPR1a* in Shennong 9819 was higher than that in Koshihikari at 72 h and lower than Koshihikari at other inoculation time points. The expression of *OsPR1b* was upregulated in Shennong 9819, and was always higher than that in Koshihikari. We detected that the gene expression changes of transcription factors *OsWRKY24* (LOC_Os01g61080), *OsWRKY30* (LOC_Os08g38990), *OsWRKY53* (LOC_Os05g27730), and *OsWRKY70* (LOC_Os05g39720) in Shennong 9819 were greater than that in Koshihikari at 36 h after inoculation (Table S6). The expression of *OsWRKY30* in Shennong 9819 was always higher than that in Koshihikari (Fig. 5), and the expression of *OsWRKY70* in Koshihikari was higher than that in Shennong 9819 (Fig. 5).

**Fig. 6:**
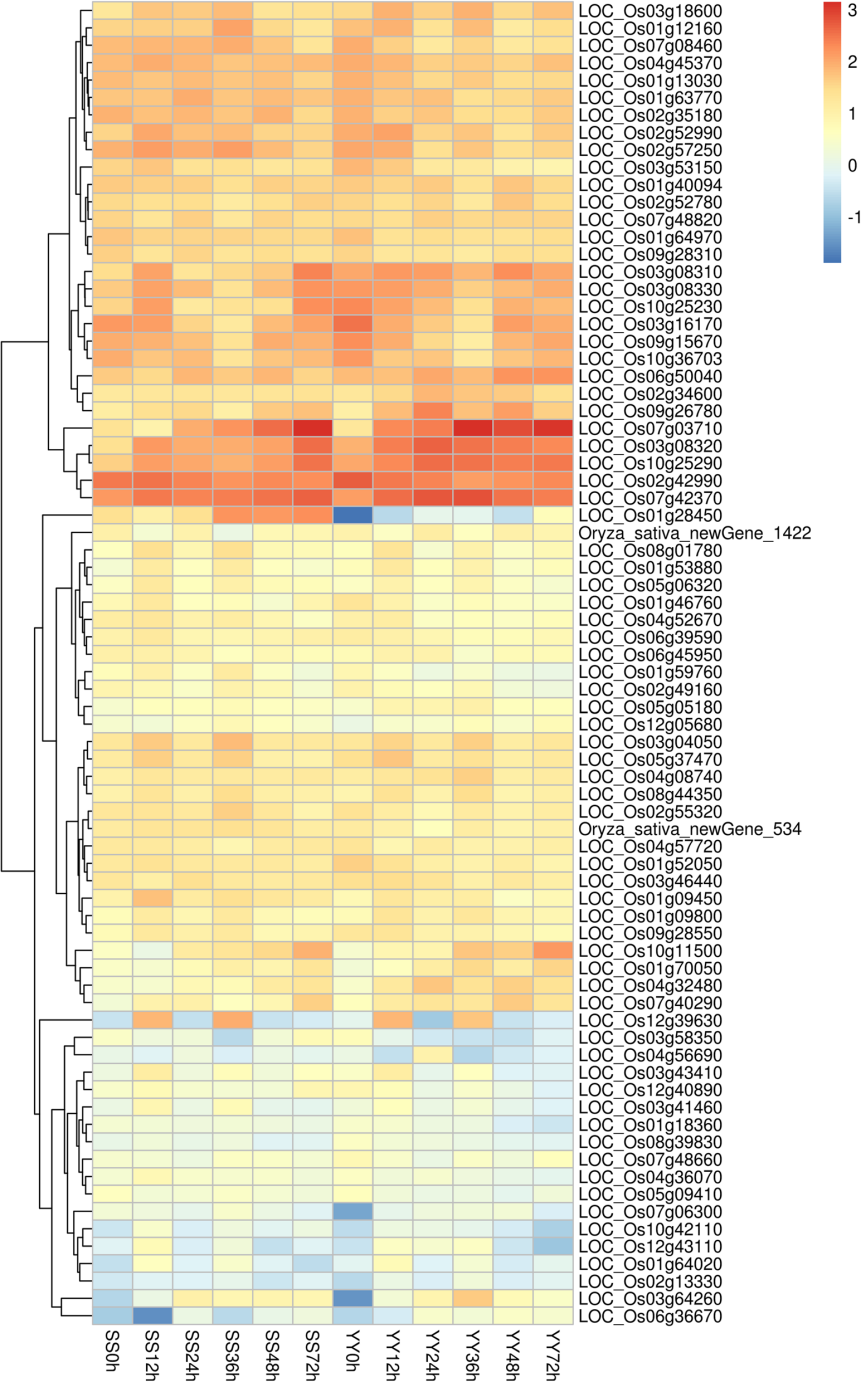
Expression profiles of genes related to plant hormone signal transduction pathways

Furthermore, we compared the DEGs involved in the plant hormone signalling pathways between the two cultivars. We found that the expression of genes closely related to plant hormone signal transduction in the leaf sheaths of the two cultivars was similar to that of phenylalanine metabolism. At 12 and 36 h, the expression levels of genes related to plant hormone signal transduction in the leaf sheaths of Shennong 9819 were significantly higher than those in the leaf sheaths of Koshihikari (Fig. 6). The expression levels of resistance-related protein kinases *OsSAPK9* (LOC_Os12g39630) [40] and *OsSAPK10* (LOC_Os03g41460) [41] were upregulated in the leaf sheaths of Shennong 9819 and were higher than those in Koshihikari (Fig. 5). In addition, the NONEXPRESSOR OF *PR1* (*NPR1*) (LOC_Os01g09800*)*, a positive regulator related to resistance, was also detected, and its expression was upregulated in both cultivars (Fig. 5). The *NPR1* homologous gene NPR3 and the transcription factor TGA2 were detected in this study. The negative regulator *OsNPR3* (LOC_Os03g46440) was downregulated in the susceptible cultivar Koshihikari at 36 h, whereas the negative regulator rTGA2.1 (LOC_Os07g48820) was downregulated in the resistant cultivar at 12 h and 36 h (Fig. 5).

As previously mentioned, the two cultivars participated in similar metabolic pathways after inoculation with *R. solani*; however, the upregulated differential expression pathways were different. Among the pathways related to disease resistance, plant hormone signal transduction and phenylalanine metabolism pathways were significantly enriched in Shennong 9819, except in Koshihikari. However, ascorbate and aldarate metabolism and linoleic acid metabolism were significantly enriched in Koshihikari, though not in Shennong 9819. It is necessary to further study the differentially expressed genes in the metabolic pathway to understand the different resistance mechanisms of the two cultivars after inoculation with *R. solani*.

### Validation of DEGs by quantitative RT-PCR (qRT-PCR)

Real-time quantitative PCR (qRT-PCR) was used to validate RNA-seq data. *OsPR1b* and other genes were selected for further validation [42]. The *C*t values obtained by qRT-PCR were normalised. The fold change in gene expression of the two rice cultivars inoculated with *R. solani* was calculated. The results showed that the expression trend of qRT-PCR was consistent with that of the RNA sequence, indicating that Illumina data were relatively reliable (Fig. S4).

## Discussion

### Gene expression changes in plants under the stress of *Rhizoctonia solani*

In the early stage of *R. solani* infection, the expression of DEGs in the inoculated leaf sheath of the resistant cultivar Shennong 9819 showed fluctuations. The number of DEGs observed in the leaf sheath inoculated with *R. solani* in Shennong 9819 was 2403 at 12 h, 190 at 24 h, 2127 at 36 h, 790 at 48 h, and 1120 at 72 h. The number of DEGs in the leaf sheath inoculated with *R. solani* in the susceptible cultivar Koshihikari first increased and then decreased; furthermore, the number of DEGs was significantly higher than that in the resistant cultivar. This is slightly different from the results reported by Zhang et al. [39]. Through a study on the early response to *R. solani* in inoculated leaves of the resistant indica rice cultivar Teqing and the susceptible japonica rice cultivar Lemont, Zhang found that the number of DEGs in inoculated leaves of both resistant and susceptible cultivars first increased and then decreased [39]. The responses were consistent with our results that the number of DEGs in the susceptible cultivar was significantly higher than that in the resistant cultivar. However, Kumari et al. [43] found that the number of DEGs in the resistant cultivar was slightly higher than that in the susceptible cultivar. These differences could be attributed to the use of different rice materials. The resistant cultivar Teqing selected in Zhang’s study is an indica rice cultivar developed in Southern China, whereas the susceptible cultivar Lemont is a conventional japonica rice cultivar developed in the United States. However, all the resistant and susceptible cultivars selected by Kumari are indica rice cultivars. We selected the japonica rice cultivar in Northeast China as the resistant cultivar and the Japanese japonica rice cultivar as the susceptible cultivar. In our study, the susceptible cultivar was more sensitive in the face of *R. solani* stress, while the differential gene expression of the resistant cultivar showed fluctuations.

### Plant–pathogen interaction

PR proteins are markers of plant defence responses related to plant resistance to pathogens [44, 45]. PR1 proteins are the first pathogenesis-related proteins identified in the PR family [46]. In this study, *OsPR1b* and *OsPR1a* were detected, and their expression trends were quite different (Fig. 5). The expression level of *OsPR1b* in the resistant cultivar Shennong 9819 was always higher than that in the susceptible cultivar Koshihikari. In contrast, the expression level of *OsPR1a* in Koshihikari was higher than that in Shennong 9819 at most time points, indicating that they may have different resistance mechanisms.

After pathogen infection, timely transcriptional regulation of defence genes in plants is crucial [47, 48]. Proteins of the WRKY family are important regulators of this defence response pathway [49, 50]. The transcription factor WRKY30 participates in the expression of genes involved in the salicylic acid (SA) and JA immune signalling pathways; furthermore, it can enhance plant resistance to biotic and abiotic stresses [51]. Previous studies have shown that *Magnaporthe grisea* induced overexpression of *OsWRKY30* is regulated by SA and/or JA [52], which can improve rice resistance to sheath blight and rice blast [53, 54]. In Arabidopsis, overexpression of *WRKY30* can enhance its resistance to *Peronospora parasitica* and cucumber mosaic virus (CMV) [55, 56]. This is consistent with the results of our study. In the present study, the expression of *OsWRKY30* in the resistant cultivar Shennong 9819 was always higher than that in the susceptible cultivar Koshihikari. This also verified that OsWRKY30 played an important role in improving the resistance of rice to *R. solani*. In addition, overexpression of *OsWRKY30* in rice significantly increased drought tolerance [57]. OsWRKY70 is a transcription inhibitor of *PR1* [58]. In the present study, expression of *OsWRKY30* in the susceptible cultivar Koshihikari was higher than that in the resistant cultivar Shennong 9819. The lower resistance of Koshihikari to *R. solani* compared with the resistant cultivar Shennong 9819 may be related to the overexpression of the transcription factor *OsWRKY70*.

### Plant signal transduction in plant disease resistance

In the natural environment, plants are constantly subjected to abiotic and biotic stresses, such as drought, salinity, and pathogen infection. Stress signals are recognised and transmitted to different cell compartments via specific signalling pathways, of which protein kinases and phosphatases are key components [59, 60]. Members of the sucrose nonfermenting1-related protein kinase2 (SnRK2) gene family are plant-specific serine/threonine kinases involved in plant responses to abiotic stresses [40, 61]. All members of the SnRK2 protein kinase gene family encoded by the rice genome are activated by hyperosmotic stress and have been designated as stress-activated protein kinases (SAPKs). We detected the upregulated resistance-related protein kinases *OsSAPK9* and *OsSAPK10* in the resistant cultivar Shennong 9819. Their expression in the resistant cultivar Shennong 9819 was higher than that in Koshihikari. Previous studies have shown that in rice plants carrying the non-host resistance gene *Rxo1*, the expression of *OsSAPK9* was significantly upregulated after infection with *Xanthomonas oryzae* pv. *oryzicola* [40] In addition, *OsSAPK9* has a positive regulatory effect on resistance to bacterial blight in rice [62]. Furthermore, it has been reported that *OsSAPK10-*mediated phosphorylation on Thr 129 of *WRKY72* weakened its DNA binding ability with AOS1, promoted the endogenous JA level of rice, and enhanced the resistance to bacterial blight [41]. These results are consistent with the results of the present study. *OsSAPK9* and *OsSAPK10* play a role in improving the disease resistance of rice in the early stages of pathogen infection. In addition, overexpression of *OsSAPK9* can significantly improve crop drought resistance [63] and NH^+^ tolerance [64]. Overexpression of *OsSAPK10* can promote the growth of root hair [65] and induce closure of stomata [66] in rice.

*NPR1* functions as a master regulator of SA signalling and plays an essential role in plant immunity [58]. Previous studies have confirmed that the overexpression of *NPR1* leads to increased host resistance to various pathogens [67, 68]. In the present study, *OsNPR1* was detected in the two cultivars; furthermore, the expression of *OsNPR1* was upregulated in both cultivars. This indicated that both cultivars initiated the *NPR1* resistance mechanism to resist infection from pathogens. *rTGA2.1* is a negative regulator of the plant defence response [69]. At 12 and 36 h, the expression of *rTGA2.1* was downregulated in the resistant cultivar. This result is consistent with previous findings [69]. Transcription factor *rTGA2.1* negatively regulated plant resistance, and its downregulation increased the resistance to *R. solani* in the resistant cultivar Shennong 9819. OsWRKY70 is an inhibitor of the *NPR1* induced resistance gene [70]. In this study, its expression in the susceptible cultivar Koshihikari was higher than that in the resistant cultivar Shennong 9819. This indicates that Shennong 9819 may induce plant resistance by regulating the expression of the *NPR1* gene in the early stage of *R. solani* infection.

### Expression of OsPAL in plant disease resistance

Phenylpropanoid metabolism is an important metabolic pathway in the secondary metabolism of plant disease resistance [71]. It leads to the biosynthesis of a wide range of plant natural products including hydroxycinnamic acids, flavonoids, coumarins, lignin, condensed tannins, and stilbenes, which have various biological functions as UV protectants, signal molecules, phytoalexins, and flower pigments [72]. L-phenylalanine ammonia-lyase (PAL; EC 4.3.1.5) is a key enzyme involved in phenylpropanoid metabolism [73]. The activity of PAL provides precursors for the biosynthesis of lignin and other phenolics such as SA [74], which accumulate when infected [75, 76]. PAL is an important regulatory defence gene. In the present study, at 12 and 36 h, the expression level of the phenylalanine metabolic pathway in the resistant cultivar Shennong 9819 was significantly higher than that in the susceptible cultivar Koshihikari. After inoculation with *R. solani*, six *PAL* genes were activated; the expression of *OsPAL1* and *OsPAL6* in the resistant cultivar was significantly higher than that in the susceptible cultivar. Previous studies have reported that PAL is involved in inducing plant disease resistance response [77, 78], and transcripts of the PAL gene accumulate in incompatible host pathogen combinations [79]. Transgenic tobacco with suppressed expression of PAL genes showed reduced basal resistance to *Cercospora nicotianae* [80]. In rice and cassava, overexpression of *PAL1* endows wild-type rice with resistance to rice blast [81] and cassava brown streak disease resistant cultivars with resistance to cassava brown stripe virus [82]. *OsPAL6* regulates biosynthesis of SA and lignin [29, 76]. Lignin accumulation is considered to be a response to plant incompatibility with pathogens [83], thereby increasing plant resistance to pathogens [84, 85]. The decrease in lignin content reduced the resistance of *Malus hupehensis* to *Botryosphaeria dothidea* [86]. In the present study, the overexpression of *OsPAL1* and *OsPAL6* in the resistant cultivar suggests that the resistant cultivar Shennong 9819 may improve its resistance to *R. solani* through the overexpression of PAL genes.

## Conclusion

In summary, the early transcriptome analysis of resistant and susceptible cultivars infected by *R. solani* revealed that the resistant cultivar has a conservative and unique defence mechanism. The differential expression of resistance-related genes is associated with the early resistance of rice to *R. solani*. In the early stage of *R. solani* infection, it was found that the number of DEGs in the inoculated leaf sheath of resistant and susceptible cultivars showed different regularity, and the expression of DEGs in resistant cultivars fluctuated. Phenylalanine metabolic pathway, plant hormone signal transduction pathway, and tropane, piperidine and pyridine alkaloid biosynthesis pathways were differentially expressed in response to resistance. In the early stage of *R. solani* stress, resistant cultivars initiated a response defence system involving overexpression of *PR* genes, *PAL* genes, and key transcription factors, as well as enrichment of defence-related pathways. In conclusion, this study provides new insights into the mechanism of rice resistance to *R. solani*. The data obtained in this study can be used to screen suitable candidate genes for genetic improvement of susceptible rice cultivars and the development of cultivars of japonica rice that are resistant to *R. solani*.

## Materials and methods

**Fig. 7:**
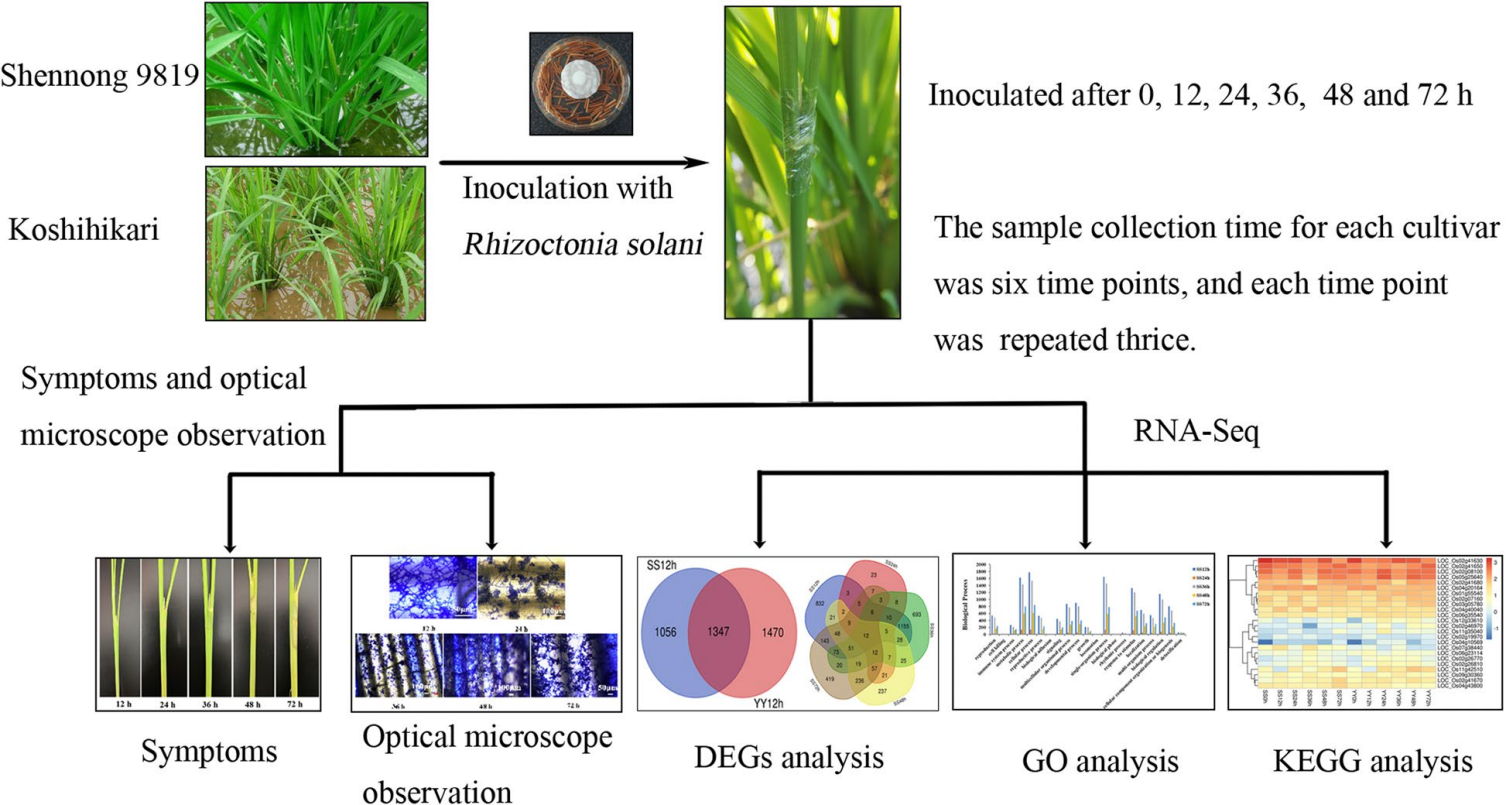
Experimental flow chart

The experimental flow chart is shown in Fig. 7.

### Plant growth

Shennong 9819, a japonica rice cultivar from the Rice Research Institute of Shenyang Agricultural University (Shenyang, China), is a rice cultivar resistant to rice sheath blight. Resistance was identified by the Rice Disease Research Office of the Shenyang Agricultural University (Shenyang, China) [19].

Koshihikari is a japonica rice cultivar from Japanese, a cultivar susceptible to rice sheath blight.

All resistant and susceptible cultivars were planted at the experimental base of Jiamus Branch of Heilongjiang Academy of Agricultural Sciences (Jiamusi, China).

### Pathogen inoculation

The strain of *R. solani* used in this experiment, R-36, was provided by the Rice Disease Research Office of Shenyang Agricultural University.

The inoculation method was carried out according to the method described by Zuo et al. [17]. A short toothpick (1 mm diameter, 1.0–1.2 cm long) colonised by R-36 was used as the inoculum for pathogen infection. Sterilised short toothpicks were placed on non-coagulated PDA plates. After the culture medium solidified, the activated mycelium was inoculated in the culture dish, cultured at 28 °C for 5 d, and a short toothpick with mycelium was selected for inoculation. At the late tillering stage, inoculation was performed using a short toothpick with mycelium. The inoculation site was the third leaf sheath from the top of the plant. Forceps were used as the inoculation tool. When inoculated, the original state of the leaf sheath should be maintained. To maintain the same temperature and humidity as the inoculated leaf sheath, the inoculated leaf sheath was wrapped with a cling film.

### Sample and method

The inoculated leaf sheaths were cut from the plants 12, 24, 36, 48, and 72 h after inoculation, and uninoculated rice leaf sheaths were collected at 0 h after inoculation as a control. The sample collection time for each cultivar was six time points, and each time point was repeated thrice. A total of 36 samples were obtained from the two cultivars. Samples collected from plants were placed in cryotubes separately, immediately frozen in liquid nitrogen, and stored at − 80 °C. The transcriptome sequencing and cDNA library construction of all samples were completed by Beijing Biomarker Technology, Inc. (Beijing, China).

### Optical microscope observation

The staining method that was used was proposed by Lux et al. [87]. Alcohol solution (95%) and glacial acetic acid were mixed in a 1:1 ratio to prepare fixative solution. Chloral hydrate (5 g) was dissolved in 2 ml distilled water to prepare saturated chloral hydrate. Aniline blue (1 g) was added to 100 ml distilled water to prepare the aniline blue staining solution.

At 12, 24, 36, 48, and 72 h after inoculation, the inoculated leaf sheaths were cut for microscopic observation. The leaf sheath tissue inoculated with *R. solani* was cut into small blocks (3 × 5 mm) with a bimodal blade. The small blocks were fixed in the fixative for 24 h and then soaked in saturated chloral hydrate aqueous solution for transparency. After the tissue was transparent, it was removed, washed with water, stained with aniline blue staining solution for 5–10 min, examined with a microscope, and photographed.

### Genome sequence and expression

Raw reads in fast format were first processed using internal Perl scripts. Clean reads were obtained by removing the reads containing adapter, poly-n, and low-quality reads from the raw reads. Q20, Q30, GC-content, and sequence duplication levels of the clean reads were calculated. All downstream analyses were based on high-quality, clean reads. These clean reads were mapped to the reference genome sequence. The reference genome used was Nipponbare MSU_v7.0. The programme Hisat2 was used to compare the reads [88], and stringties were used to assemble, evaluate, and quantify the reads [89]. Fragments per kilobase of transcript per million fragments mapped (FPKM) was used to calculate gene expression [90].

### Screening and functional annotation of DEGs

Differential expression analysis of the differential groups was performed using DEseq [91]. The resulting *P* values were adjusted using Benjamini and Hochberg’s approach to control the false discovery rate. Genes with an adjusted *P*-value < 0.01 found by DEseq, were assigned as differentially expressed. The GO enrichment analysis of the DEGs was implemented using the GOseq R package [92], which can adjust for gene length bias in DEGs. We used KOBAS software to test the statistical enrichment of DEGs in the KEGG pathways [93].

### Real-time PCR analysis

Eight genes that were co-expressed in the two cultivars were selected for real-time quantitative PCR. Specific primers were designed using Primer-BLAST of NCBI and are listed in Table S7.

The RNA was extracted using the TaKaRa MiniBEST Plant RNA Extraction Kit, and the 1st Strand cDNA Synthesis Kit (TaKaRa, Tokyo, Japan) was used for reverse transcription into cDNA. qRT-PCR experiments were performed using a Bio-Rad CFX96 Real-Time PCR System (Bio-Rad, Hercules, CAUSA) according to the manufacturer’s instructions. Reactions were prepared using 20 μL of total volume, 10 μL of SYBR® Premix Ex Taq™ II, 1.0 μL of gene-specific primers (0.5 μL each primer), and 0.5 μl of cDNA.

The reaction procedure was run as follows: (1) 95 °C for 30 s; (2) 95 °C for 5 s, 60 °C for 30 s, for 40 cycles. (3) 95 °C for 15 s, 60 °C for 60 s, and 95 °C for 15 s. The actin gene was used as an internal control to normalise the data. Gene expression levels were calculated using the 2^-ΔΔCT^ algorithm [94].

## Supplementary Information

**Additional file 1: Fig. S1.** Symptoms of sheath blight disease detected in Shennong 9819 and Koshihikari.

**Additional file 2: Fig. S2.** Infection of hyphae in inoculated leaf sheaths at different time points.

**Additional file 3: Fig. S3.** Overall relatedness of transcriptomes at different times. (SS for Shennong 9819; YY for Koshihikari).

**Additional file 4: Fig. S4.** RT-qPCR validation of differentially expressed genes identified by Illumina sequencing. Histogram: Relative expression, detection results of real-time fluorescent quantitative PCR; Line graph: log2FC, fold change in differentially expressed genes in the transcriptome.

**Additional file 5: Table S1.** Statistics of Illumina sequencing data. (SS for Shennong 9819; YY for Koshihikari).

**Additional file 6: Table S2.** Summary for clean read mapping to the *Oryza sativa* Nipponbare reference genomeclean reads. (SS for Shennong 9819; YY for Koshihikari).

**Additional file 7: Table S3.** The 2275 co-regulated genes in both rice cultivars at different time points (SS for Shennong 9819; YY for Koshihikari).

**Additional file 8: Table S4.** Analysis of pathways involving upregulated genes after inoculation with *Rhizoctonia solani* in Shennong 9819. (SS for Shennong 9819).

**Additional file 9: Table S5.** Analysis of pathways involving upregulated genes after inoculation with *Rhizoctonia solani* in Koshihikari. (YY for Koshihikari).

**Additional file 10: Table S6.** DEGs associated with plant–pathogen interaction.

**Additional file 11: Table S7.** Specific primers for differential gene sequences for qRT-PCR.

## Data Availability

All datasets generated or analysed during this study are included in this published article and its supplementary information files.

## Abbreviations

DEGs: Differentially expressed genes
ET: Ethylene
GO: Gene ontology
JA: Jasmonic acid
KEGG: Kyoto encyclopaedia of genes and genomes
*NPR1*: *Nonexpressor of PR1* genes
PAL: L-phenylalanine ammonialyase
PG: Polygalacturonase
PGIP: Plant polygalacturonase-inhibiting protein
PR: Pathogenic-related
qRT-PCR: Real-time quantitative PCR
RNA-Seq: RNA-sequencing
SA: Salicylic acid
SAR: Systemic acquired resistance
TPM: Transcripts per million
*Xoo*: *Xanthomonas oryzae* pv. *oryzae*
*Xoc*: *Xanthomonas oryzae* pv*. Oryzicola*
