# Supplementary figures and images for: Gene expression analysis of resistant and susceptible rice cultivars to sheath blight after inoculation with Rhizoctonia solani
Source: BMC Genomics. 2022 Apr 7;23:278. doi: 10.1186/s12864-022-08524-6 (PMC8991730; doi:10.1186/s12864-022-08524-6)

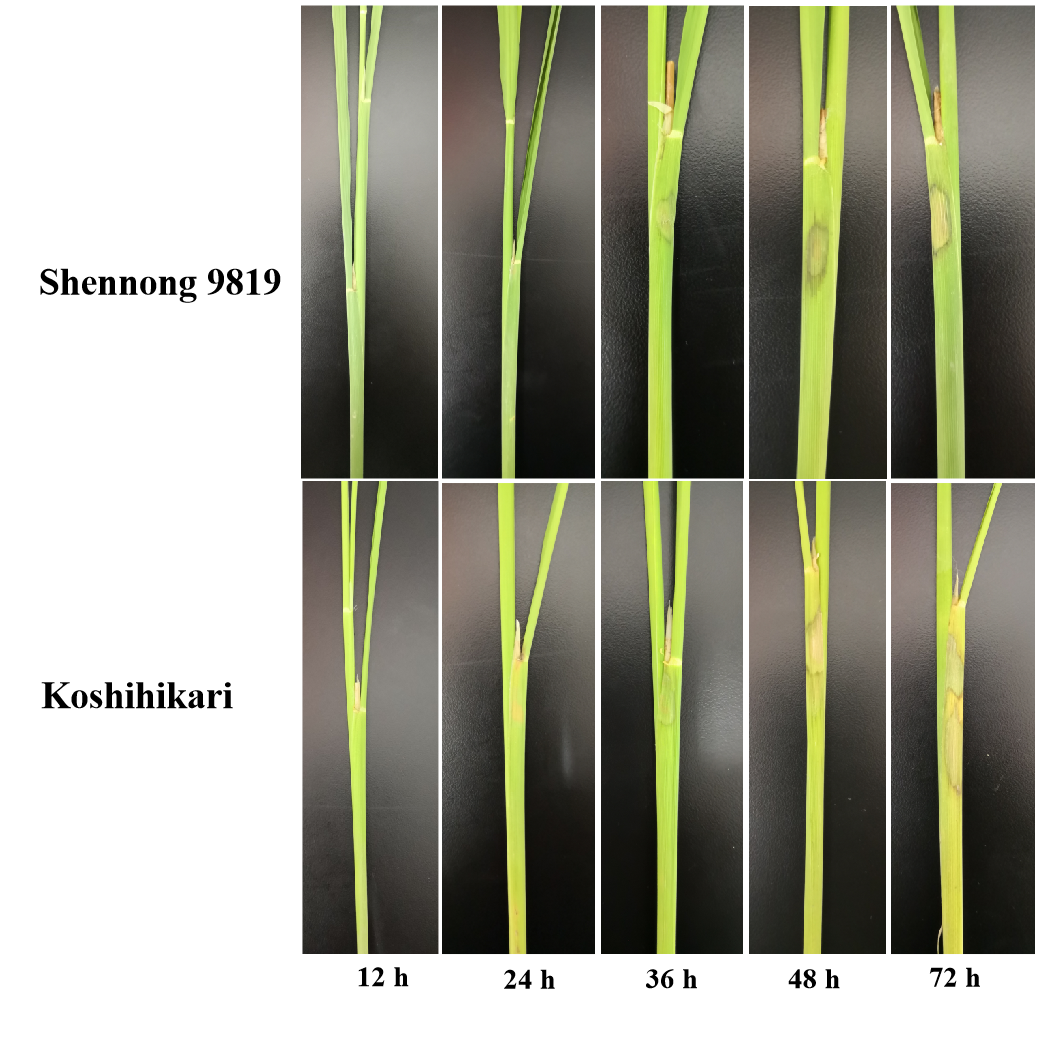

Supplement: Supplementary file 1 — Additional file 1: Fig. S1. Symptoms of sheath blight disease detected in Shennong 9819 and Koshihikari. [file 12864_2022_8524_MOESM1_ESM.tif]

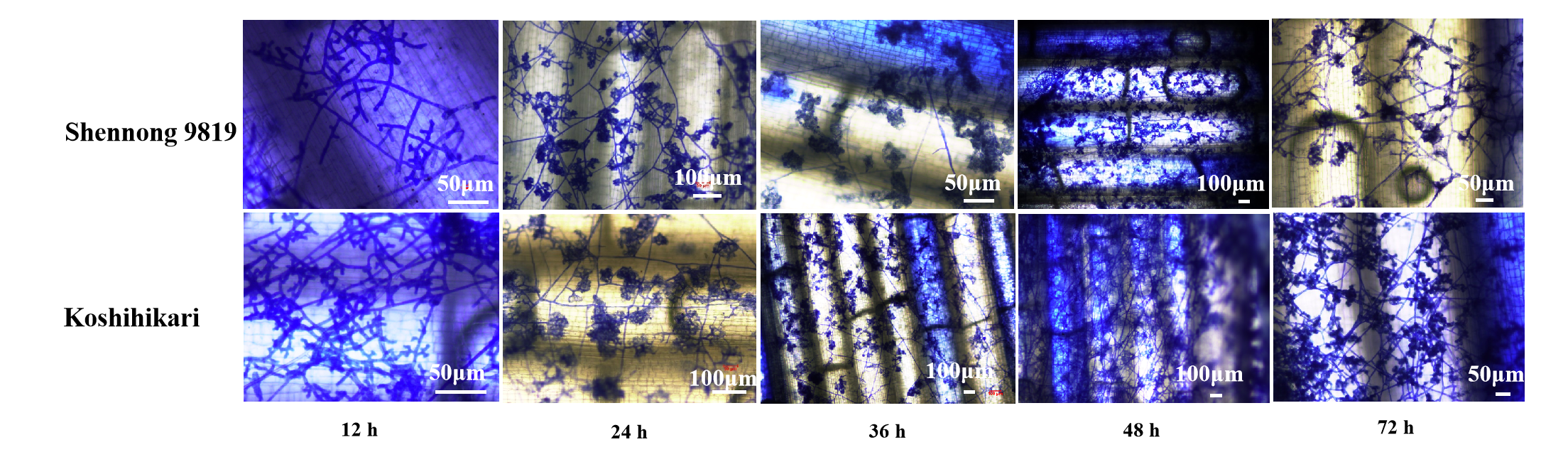

Supplement: Supplementary file 2 — Additional file 2: Fig. S2. Infection of hyphae in inoculated leaf sheaths at different time points. [file 12864_2022_8524_MOESM2_ESM.tif]

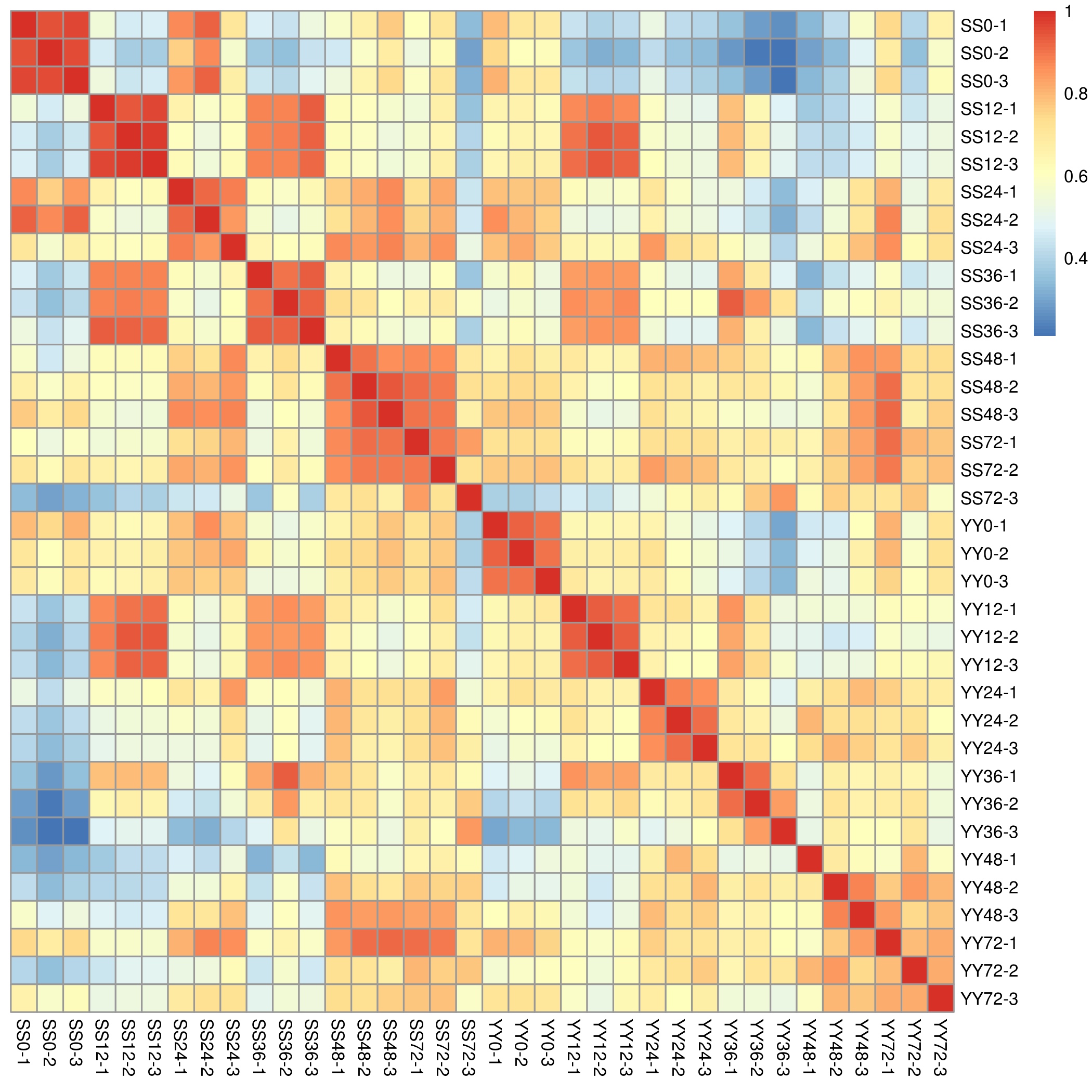

Supplement: Supplementary file 3 — Additional file 3: Fig. S3. Overall relatedness of transcriptomes at different times. (SS for Shennong 9819; YY for Koshihikari). [file 12864_2022_8524_MOESM3_ESM.png]
