# Supplementary material for: Gene expression analysis of resistant and susceptible rice cultivars to sheath blight after inoculation with Rhizoctonia solani
Source: BMC Genomics. 2022 Apr 7;23:278. doi: 10.1186/s12864-022-08524-6 (PMC8991730; doi:10.1186/s12864-022-08524-6)
Supplement: Supplementary file 4 — Additional file 4: Fig. S4. RT-qPCR validation of differentially expressed genes identified by Illumina sequencing. Histogram: Relative expression, detection results of real-time fluorescent quantitative PCR; Line graph: log2FC, fold change in differentially expressed genes in the transcriptome. [file 12864_2022_8524_MOESM4_ESM.pdf]

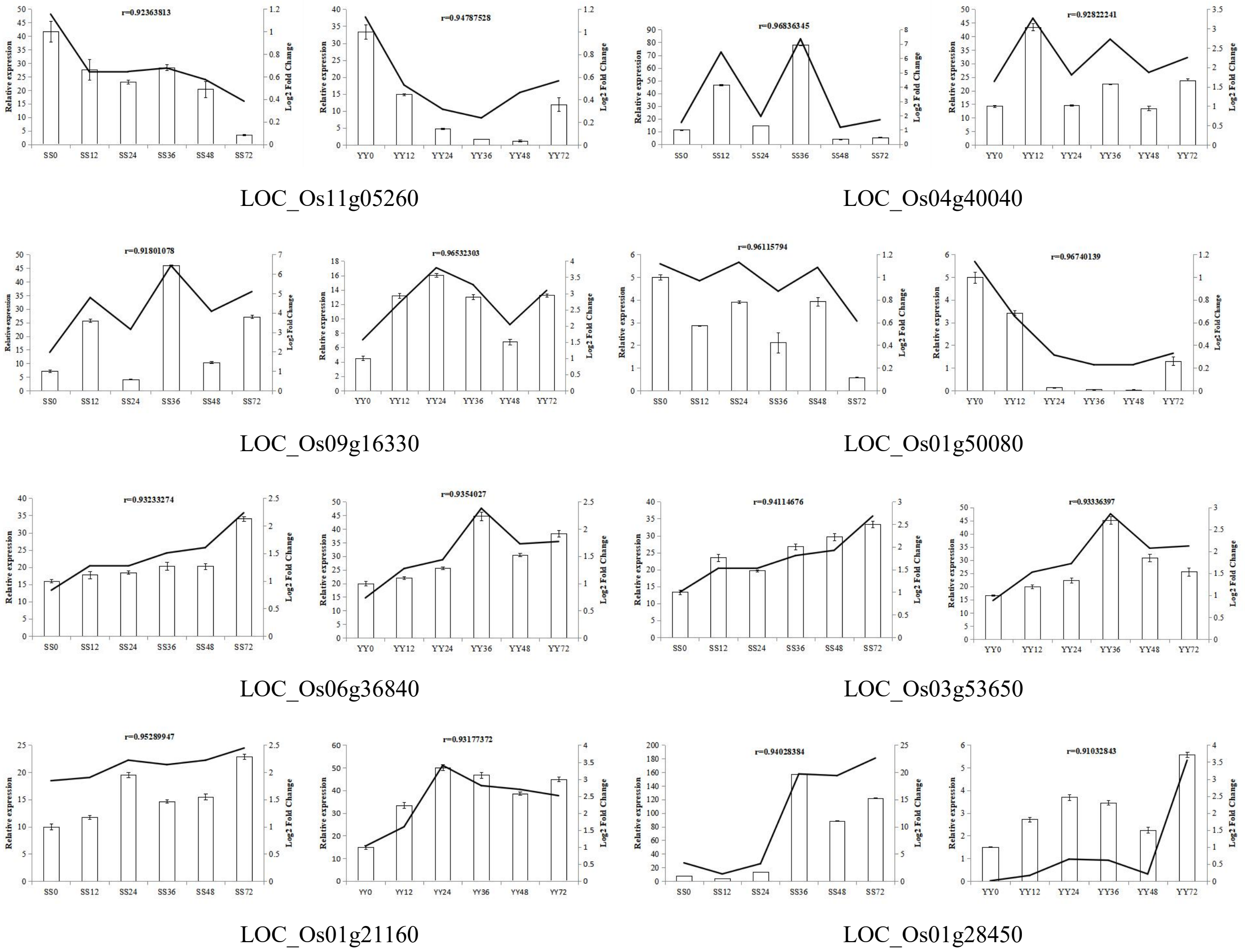

Figs.4 RT-qPCR validation of parts of differentially expressed genes identified by Illumina sequencing.

Histogram: Relative expression, detection results of real-time fluorescent quantitative PCR;

Line graph: log2FC, fold change in differential expression genes in the transcriptome.

SS0, SS12, SS24, SS36, SS48, and SS72 represent relative expression and log2FC fold change in DEGs in transcriptome from leaf sheath of Shennong 9819 at 12, 24, 36, 48, and 72 h after inoculation; YY12, YY24, YY36, YY48, and YY72 represent relative expression and log2FC fold change in DEGs in transcriptome from leaf sheath of Koshihikari at 12, 24, 36, 48, and 72 h after inoculation.
